# The Operative Treatment of Retroversion and Retroflexion of the Uterus

**Published:** 1903-12-12

**Authors:** 


### THE OPERATIVE TREATMENT OF RETROVERSION AND RETROFLEXION OF THE UTERUS.

Much of the failure which is met with in the treatment of retroversion and retroflexion of the uterus is due, Dr. Young [1] believes, to the want of a clear understanding of the complex nature of the condition, which does not consist simply of malposition of the fundus. In reviewing the anatomy of the uterus Dr. Young calls attention to the importance of the utero-sacral ligaments which suspend the uterus from the under surface of the second sacral vertebra. These ligaments are very strong and are unaffected by pregnancy or delivery. Next to these in importance, as suspensory ligaments, are the broad ligaments. These and also the round ligaments are stretched and rendered lax by pregnancy. The latter are not suspensory in their action in the least degree. They hold forward the fundus, but have no influence on the lower segment of the uterus. Traction on them tends to lower the body of the uterus. Normally the fundus can be retroflexed with ease, but displacement of the lower uterine segment is prevented by the utero-sacral ligaments.

The conditions found when retroflexion or retroversion are present are laxity of the broad and round ligaments; stretching of the utero-sacral ligaments (if retroversion is present); laxity of the pelvic fascia and levatores ani allowing prolapse of the pelvic diaphragm; chronic congestion of the uterus. Any method applied for the relief of retroversion and retroflexion must depend upon a consideration of all the structures involved in the deformity of a given case and the degree of their involvement. The ideal procedure, in Dr. Young's opinion, is as follows: curettage (since endometritis is always present), repair of injuries to the cervix, retro-vaginal septum, and anterior wall of the vagina. Laparotomy and shortening of the utero-sacral ligaments. Shortening of the round and broad ligaments. The disadvantages are the length of time necessary to complete the operation, and the difficulty in its performance in fat subjects. An alternative method is ventral suspension, which, when performed in combination with other procedures, does relieve the malposition and prevent its recurrence. Its dangers, when properly performed, are small, if any, in subsequent pregnancies. Its advantages are the ease and rapidity with which it may be effected. The operation consisting, in brief, of suturing the fundus of the uterus to the anterior abdominal wall by a couple of catgut sutures.

Ventral fixation is applicable only when the patient has passed the child-bearing period.

Non-operative measures are successful in some uncomplicated cases, and depend for their value upon relief of pelvic congestion, the restoration of normal circulation, and the return of normal elasticity and tone to the ligaments and muscular structures.

[1] Medical Record, Oct. 24.

